# ^60^Co-γ Irradiation Affects the Enzymatic Antioxidant System of the Citrus Red Mite *Panonychus citri* (Acari: Tetranychidae)

**DOI:** 10.3390/molecules19056382

**Published:** 2014-05-19

**Authors:** Ke Zhang, Zhibin Li, Shaowen Zhu, Qunfang Weng

**Affiliations:** Key Laboratory of Pesticide and Chemical Biology, Ministry of Education, Guangzhou 510642, China; E-Mails: zhangke2110@163.com (K.Z.); zhibinli00@163.com (Z.L.); chuk_shiuman@163.com (S.Z.)

**Keywords:** ^6^^0^Co-γ irradiation, antioxidant enzymes, *Panonychus citri*

## Abstract

Radio-(^6^^0^Co), which emits γ rays, has been used worldwide in pest control. The aim of the present study was to analyze the effect of effective-low-power ^6^^0^Co-γ irradiation on the enzymatic antioxidant system of the citrus red mite *Panonychus citri*. One day old female adults were exposed to 0.4 kGy ^6^^0^Co-γ irradiation and on the, 6th h, 1st day, 2nd day, and 5th day post treatment, the mites were euthanized for biochemical analysis. The activity of superoxide dismutase (SOD), catalase (CAT), peroxidase (POD), phenoloxidase (PO) and acetylocholinesterase (AchE) were investigated. POD and CAT activities, as well as SOD were higher in the irradiated mites. We found that exposure to ^6^^0^Co-γ radiation resulted in increased activities of SOD, CAT, POD and decreased AchE activity. When the recovery time lasted till the 5th day, the activities of POD and PO were significantly lower than the control, whereas the SOD, CAT and AchE activities returned to control levels. Cells possess protein repair pathways to rescue oxidized proteins and restore their functions, but if these repair processes fail, oxidized proteins may become cytotoxic. Our results confirm the hypothesis that low dosages of ^6^^0^Co-γ irradiation increase the level of oxidative stress in *P. citri* adults in a short time, causing meanwhile damage and sterility. The results of this study also indicate that stress caused by exposure to irradiation could inhibit the cholinergic system in *P. citri*.

## 1. Introduction

In many organisms, with ^60^Co-γ irradiation free radicals in the body, such as singlet oxygen, hydrogen peroxide, hydroxyl radical and superoxide anion will increase. These free radicals interact with biological molecules and hydrogen addition, electron trapping, hydrogen transfer, polymerization, decomposition and dismutation reactions damage these biomolecules. The endogenous enzymatic antioxidant system is important to protect the organism against high concentrations of ROS. This system is composed mainly by the enzymes superoxide dismutase (SOD), catalase (CAT), and peroxidases (POD) [1,2]. Superoxide dismutase detoxifies O_2_^−^, catalase and peroxidases, which metabolize cellular H_2_O_2_. Oxidative damage to proteins can range from specific amino acid modifications and peptide scission to loss of enzyme activity [3,4]. It has been demonstrated that the activities of antioxidant enzymes in insects can be induced by some external factors [5,6,7]. It is well known that oxidative stress can induce apoptosis, however, antioxidants (CAT, SOD, POD) usually prevent tissue damage under normal conditions. Already in 1991, it was shown that hydrogen peroxide is able to induce apoptosis, which is prevented by catalase. CAT also prevented spontaneous apoptosis, suggesting that the generation of H_2_O_2_ might be an important trigger mechanism responsible for the short lifespan of mature neutrophils [8,9]. In this study, the protective effect of melanins synthesized by the action of the polyphenol oxidase (PO) and acetylocholinesterase (AchE) that terminates neurotransmission by hydrolyzing the acetylcholine released by the motoneurons at the neuromuscular junctions was assessed. 

The citrus red mite, *Panonychus citri* (Acari:Tetranychidae), is a worldwide citrus pest with more than 80 host plants such as citrus, rose, almond, pear, castor bean, and several broadleaf evergreen ornamentals [10,11]. It has a short life cycle and high reproductivity. It is also an important sensitizing allergen to asthma and rhinitis among people around citrus orchards [12,13,14]. Irradiation as a quarantine treatment to control pests is accepted by most governments and countries. Gamma rays and electron beams are under investigation and even used in some parts of the world to prevent development or reproduction of the arthropod pests (mites and insects). Use of irradiation is considered an appropriate quarantine treatment due to its beneficial aspects. It does not produce undesirable residues, has a short treatment time compared with fumigations and is economically comparable with other methods [15]. Furthermore, irradiation is applicable to packaged products [16] and could be combined with complementary treatments such as low temperature which is especially applicable for commodities requiring fast shipment [17]. Irradiation differs from other commercial treatments in that the end point of the treatment is not an acute mortality but prevention of further biological development and reproduction [18].

For insects, it is generally accepted that radiotolerance increases as they develop with some exceptions [19]. Irradiation for postharvest disinfestation has been investigated for various fruits and vegetables and shows great promise in that it sterilizes insects at doses that are low enough not to be detrimental to most fruits and vegetables [20,21]. Although the same hypothesis is expected on mites, the mechanisms has not been studied. Specifically, for spider mites the effect of irradiation on its enzyme activity has not been studied yet. Our previous study proved that 0.4 kGy could prevent the propagation of *P. citri*, and besides, 0.4 kGy did not affect the nutrient composition of citrus. Considering that a previous study conducted in our laboratory showed that the adults were the most radiation-tolerant stage and the gamma irradiation at the dosage 0.4 kGy made citrus red mites at different stages die or sterile [22]. The aim of the present work is to supply evidence on the interference of enzymes in the oxidative stress as a response to irradiation exposure. The purpose of this study was to examine the impact of irradiation at a general dose produced by a commercial device on the activities of a range of antioxidant enzymes in *P. citri*, trying to better understand the radiation mechanism of action that may be involved with the positive results of irradiation on sterility for pest control. The most important studies on mites are reviewed in the following paragraphs.

## 2. Results and Discussion

**Figure 1:**
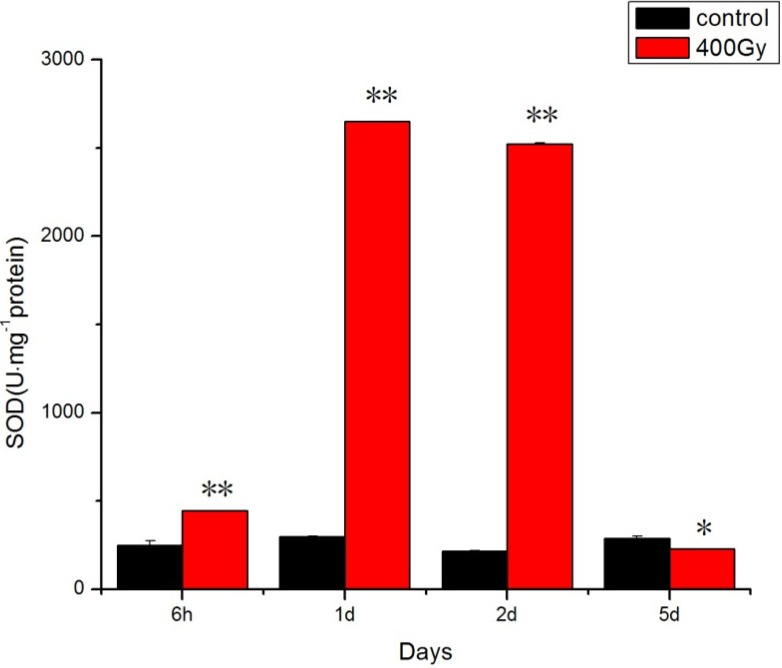
Effects of ^6^^0^Co-γ irradiation on SOD activity of *P. citri* adults for different lengths of time. Values are mean ± SD (*n* = 3). Asterisk designates statistically significant difference between control and irradiated adults (** *p* < 0.01; * *p* < 0.05).

In the present study, the change in SOD activity suggested that ^6^^0^Co-γ irradiation induces superoxide radicals in *P. citri* adults. This has also been found in *H. armigera* adults [23]. It has been reported that an increase in SOD activity is probably a response to increased ROS generation [24]. However, longer recovery times (5th day) resulted in inhibition of SOD activity in comparison with the adults at day 1, and the activity reverted to the control level (Figure 1). 

That is consistent with previous reports showing that high doses of irradiation suppress the activity of protective enzymes, such as SOD in normal cells [25,26]. Irradiation is generally considered to be a phytosanitary treatment for animals. Radiation is known to lead to the production of ROS. This is due to photooxidation reactions by endogenous photosensitizers since nucleic acid bases absorb radials [27,28]. The goal of quarantine or phytosanitary treatments is to prevent the invasion and propagation of regulated pests [29]. Phytosanitary treatments, allow for the destruction or removal of pests or for making the pest reproductively sterile, to achieve the goal of pest control. In 2006, the USDA-APHIS approved generic treatments of 0.15 kGy for fruit flies and 0.40 kGy for all insects except pupa and adult stage Lepidoptera [30]. However, no previous studies were conducted to elucidate the effect of ^6^^0^Co-γ irradiation upon the antioxidant system in mites. Thus the present study represents a pioneering trial for establishing the potentiality of these compounds in causing infertility in *P. citri* and showed that minimum-effective (0.4 kGy) ^6^^0^Co-γ irradiation can increase the antioxidant enzyme activity in a short time. 

**Figure 2:**
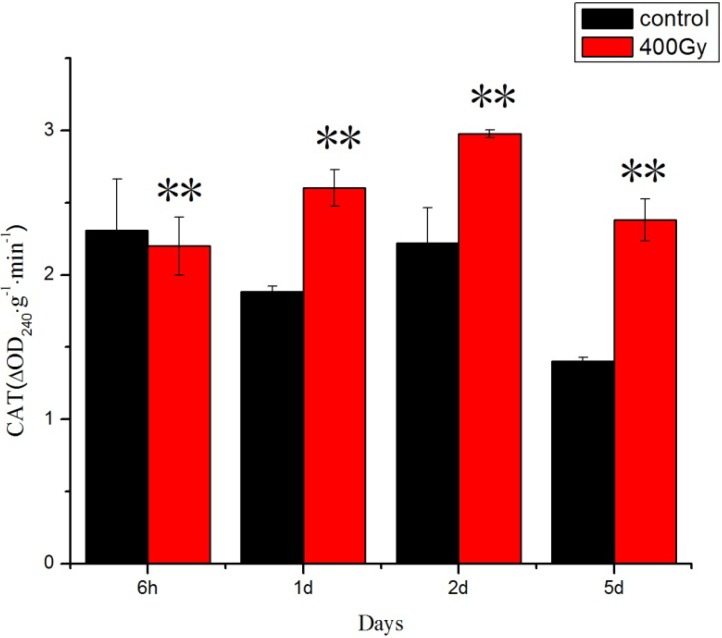
Effects of ^6^^0^Co-γ irradiation on CAT activity of *P. citri* adults for different lengths of time. Values are mean ± SD (*n* = 3). Asterisk designates statistically significant difference between control and irradiated adults (** *p* < 0.01).

**Figure 3:**
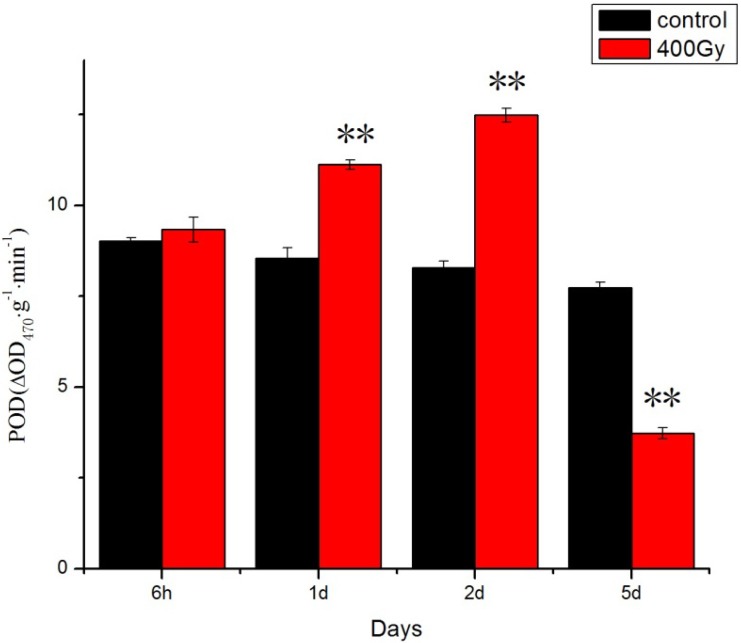
Effects of ^6^^0^Co-γ irradiation on POD activity of *P. citri* adults for different lengths of time. Values are mean ± SD (*n* = 3). Asterisk designates statistically significant difference between control and irradiated adults (** *p* < 0.01).

Total antioxidant capacity is a resultant measure of the ability of all antioxidants present in an organism to counteract the oxidation of an indicator by an oxidant, or to reduce an indicator substance [31,32]. Previous studies have shown that CAT can protect against oxidative stress and extend the lifespan of insects [33,34]. In our study, CAT activity significantly increased (Figure 2) and a significant increase of POD activity in *P. citri* adults was recorded the mites that were irradiated before 2 days (Figure 3). ^6^^0^Co-γ irradiation stress to POD was severe in *P. citri* adults at longer recovery time. Meng studied the effect of UV irradiation on *Helicoverpa armigera* and found that exposure to UV light resulted in increased SOD, CAT and POD activities to decrease the level of oxidative stress in *H. armigera* adults [23]. We have confirmed that ^6^^0^Co-γ irradiation may disturb the functional activity of proteins and intensify the activity of protein oxidation processes. The induction of antioxidant enzymes as a result of ^6^^0^Co-γ irradiation may also indicate the over-production of ROS. 

**Figure 4:**
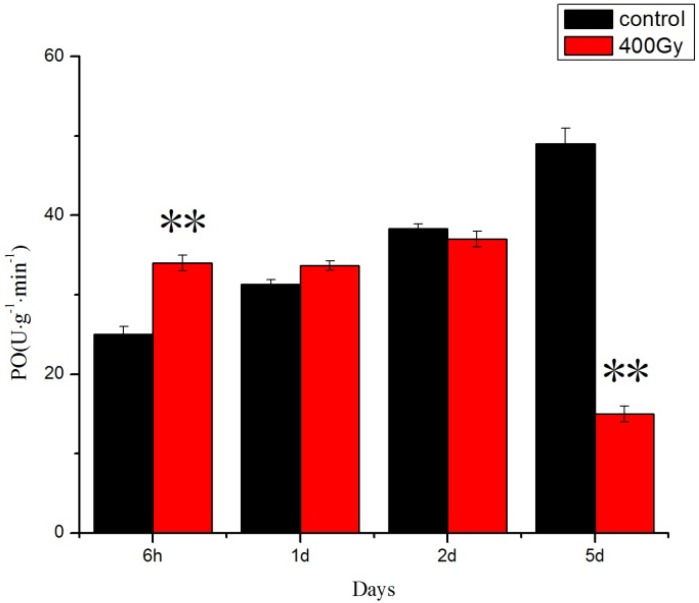
Effects of ^6^^0^Co-γ irradiation on PO activity of *P. citri* adults for different lengths of time. Values are mean ± SD (*n* = 3). Asterisk designates statistically significant difference between control and irradiated adults (** *p* < 0.01).

PO activity also increased at 6 h but shaply declined after 5 days in *P. citri* adults (Figure 4). 

The rapid enhanced activities of SOD, CAT, POD and PO in response to ^6^^0^Co-γ irradiation are a likely defense against oxidative damage due to the accumulation of ROS. These processes may be mirrored in insect physiological adaptations. However, prolonged recovery time resulted in decreased activities of SOD, CAT, POD and PO, accompanied by impaired antioxidant capacity and high levels of oxidative stress. 

**Figure 5:**
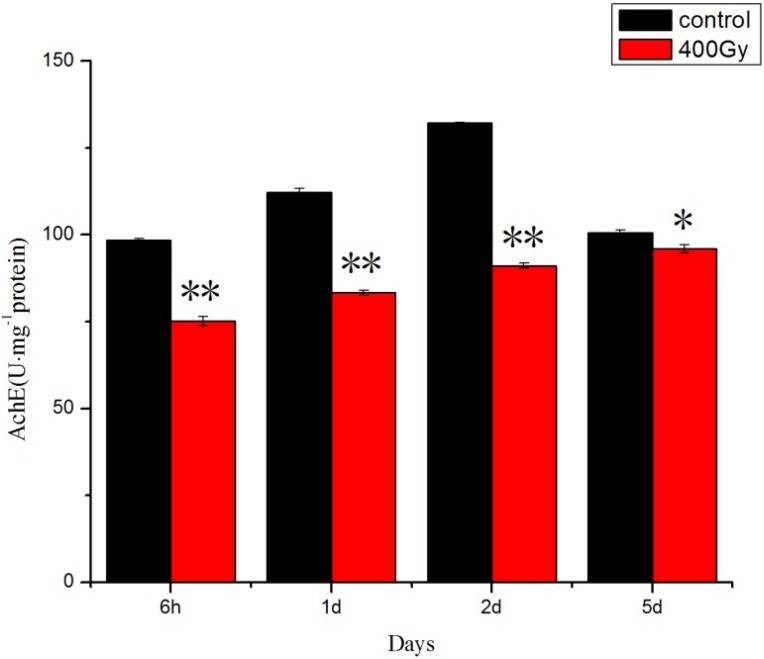
Effects of ^6^^0^Co-γ irradiation on AchE activity of *P. citri* adults for different lengths of time. Values are mean ± SD (*n* = 3). Asterisk designates statistically significant difference between control and irradiated adults (** *p* < 0.01; * *p* < 0.05).

Presently, domestic and foreign research on insect AchE and PO is concentrated in the resistance, and studies of changes to other stress conditions of two kinds of important detoxification enzymes are relatively infrequent. Our results showed that the AchE activity declined in *P. citri* adults. However, after 5 days the AchE activity reverted to the control level (Figure 5). Some researchers have reported that the AchE activity in addition to being influenced by pesticides, is also influenced by other stress factors, such as hypoxia, ultrasonic, bacterial invasion and various kinds of apoptosis-inducing factorw [35,36,37]. Wang studied the effects of X-rays on the activity of acetylcholinesterase founded that AchE activity in the 3 Gy group firstly decreased and then increased [38]. AchE activity decreased, leading to increased AchE levels [39,40]. An *in vitro* study found that when the cells enter apoptosis state there is a large amount of AchE expression [41], and during apoptosis, AchE exhibits at least two functions: an increase of AchE protein can inhibit cell growth at first, then, AchE enters the nucleus and promotes cell apoptosis [42]. Although the obtained results suggest an interaction between irradiation and the protective enzyme system, further research at a molecular scale should be conducted in order to understand the mechanism by which this interaction leads to the inactivation of these enzymes.

A significant decrease of viability in eggs layed by the adults mites pretreated with irradiation was observed at general irradiation doses that had no marked effect on citrus fruits. The present study demonstrated, for the first time, the protective role of the protective enzymes in pest mites’ exposure to irradiation, and the important role of antioxidants in maintaining the function and integrity of the body. We speculate, therefore, that irradiation treatment might have exacerbated detrimental influence on mites, however, the detailed molecular mechanism of these influences of sterility needs further study.

## 3. Experimental

### 3.1. Insects

Adults of *P. citri* were collected from citrus plants in an orchard at the South China Agricultural University, Guangzhou Province (China), and subsequently reared in a climate chamber at 20 ± 1 °C, 70% ± 10% relative humidity, and under a photoperiod of L16:D8 (L6:00–22:00; D, 22:00–6:00), reared for many generations formed for experimental population. One-day old adult mites homogenized in ice-cold buffer (5 mM phosphate pH 7.8 or pH 6.8). The seriflux was then centrifuged for 15 min at 12,000 *×g* (4 °C). The supernatant was then used in analysis.

### 3.2. ^60^Co-γ Irradiation

The experimental material was irradiated by ^60^Co–γ-rays from a Nordion (Ottawa, ON, Canada) ^60^Co–γ radiation source located at the Furui High-energy Technology Co. Ltd., (Nansha District, Guangzhou, Guangdong Province, China). Dose rate was 4.0 Gy/min in all experiments. A Fricke dosimeter was used (according to ISO/ASTM E 1026-04).

### 3.3. Analysis of Antioxidant Enzyme Activities

The activity of SOD, CAT, POD, PO, AchE and GST was determined by spectrophotometry. The protein concentration was measured using Bradford’s Coomassie Brilliant Blue G-250 [43], bovine serum albumin was used as standard. 

SOD activity was assayed by SOD enzyme kit. One unit of SOD activity was defined as the amount of enzyme required to cause 50% inhibition of the xanthine and xanthine oxidase system reaction in 1 mL enzyme extraction of 1 mg protein. SOD activity was expressed as U·mg^−1^ protein.

CAT activity was determined by measuring the decrease in absorbance at 240 nm due to H_2_O_2_ decomposition. CAT activity was expressed as ΔOD·g^−1^·min^−1^.

POD activity was determined spectrophotometrically at 470 nm by catalyzing the oxidation in the presence of H_2_O_2_ of a substrate. POD activity was expressed as ΔOD·g^−1^·min^−1^.

PO activity was assayed measuring the increase in absorbance at 420 nm using 4-methylcatechol as substrate. One unit of PO was defined as the amount of enzyme that caused the increase of one unit of absorbance at 420 nm in one min [44]. PO activity was expressed as ΔOD·g^−1^·min^−1^.

AchE activity were analyzed according to the standard spectrophotometric procedures provided with the commercial kit (KGT037, Nanjing KeyGEN BioTECH, Nanjing, China) using a UV2000 spectrophotometer, which are modified from the method of Ellman [45].

### 3.4. Statistical Analysis

Statistical analysis was conducted for each of the measured traits by analysis of variance (ANOVA) and the means were separated by Duncan Multiple Range test using the SPSS software, version 18.0 (SPSS, Inc., Chicago, IL, USA). In addition, a linear discriminant analysis (LDA) was used to assess the influence of either different times on enzyme activities. Posterior comparisons were performed for each recovery time irradiation group *vs.* sham irradiation group and *p*-values < 0.05 being considered significant. All the assays were carried out in triplicate. The results were expressed as mean values with standard deviation (SD).

## 4. Conclusions

Our results suggested that ^6^^0^Co-γ irradiation hamper the functional activity of protein and intensify the activity of protein oxidation processes. Besides, the effect of irradiation on protective enzyme system related to the processing time. The induction of antioxidant enzymes as a result of ^6^^0^Co-γ irradiation may also indicate the over-production of ROS.
